# Vaginal Hysterectomy

**Published:** 1887-11

**Authors:** Christian Fenger

**Affiliations:** Professor of Clinical Surgery in the College of Physicians and Surgeons, of Chicago


THE MEDICAL JOURNAL AND EXAMINER.

VOL. LV. NOVEMBER, 1887. No. 5.

### VAGINAL HYSTERECTOMY.

### THE ACTUAL STATUS OF THE OPERATION AND REPORT OF FOUR CASES.

BY CHRISTIAN FENGER, M. D.

PROFESSOR OF CLINICAL SURGERY IN THE COLLEGE OF PHYSICIANS AND SURGEONS, OF CHICAGO.

IN the short space of eight years, since 1879, when Czerny, Billroth, Schröder and Mikulicz revived vaginal hysterectomy, a large number of operations, probably nearly 500, have been performed, and a considerable amount of literature on this subject has been brought forth. In spite of adverse criticism, and the apparent gravity of the operation, it has had a more thorough and general trial than could have been expected in so short a time, and for an operation attended by the uncertainty as to results which pertains to all operations for malignant tumors.

Modern surgery, with its allowance of more radical and wider lines for the operations for the removal of sarcomas and carcinomas, has generally procured a larger percentage of definite cures for these tumors in all parts of the body. Although a percentage of between ten and twenty permanent cures seems small, yet it holds out a distinct ray of hope as compared with inevitable death on the side of non-interference—a ray strong enough to cause operations of this kind to be kept up, even if the immediate risk to life is considerable. This immediate mortality, however, must be within certain limits to permit of the general adoption of an operation. Freund's abdominal hysterectomy, with its mortality of seventy per centum, was too grave even for malignant tumors. Vaginal hysterectomy, with an initial mortality of about thirty per centum, was not so grave as to prevent the general adoption of the operation. But even this mortality has been one of the main arguments advanced from the beginning against the practicability of the operation. It is therefore an important question to ask: Has vaginal hysterectomy become less dangerous since its revival by Czerny in 1879?

The mortality from the operation for the first five years, up to 1884, has been stated by Mundé to be twenty-eight per centum. In a paper read before the American Gynæcological Association in 1884, he tabulated all the cases to be found in the literature both in Europe and the United States. The number of operations was two hundred and fifty-five, with seventy-two deaths. For the operations of 1885, recorded in Virchow's Jahresbericht 1886, the mortality is already much lower. I find reports from thirty-two operators of one hundred and six cases, with seventeen deaths, or sixteen per centum. R. Martin reports, in his work of 1887, sixty-six cases with eleven deaths, or sixteen per centum. Special reports from individual operators in the past year show even more favorable results. Thus we find reported by Klotz seventeen cases, with no deaths, and by T. Gaillard Thomas fifteen cases, with no deaths. Most valuable, however, from the large number of cases, are the recent reports of Fritsch and Leopold. Fritsch reports sixty cases, with seven deaths, or eleven and two-thirds per centum, and Leopold forty-eight cases, with three deaths, or six and one-quarter per centum. The reports from the last operators include all their operations from 1883 to 1887. Leopold's mortality of six and one-quarter per centum is the lowest on record for a large number of cases.

In the beginning of his work in this line, every operator has undoubtedly operated in cases which were too far advanced for reasonable hope of eradication of the disease. The more recent the origin of the tumor, and, consequently, the more limited the extent of the growth, the better are the immediate, as well as the remote, prospects for the patient. If, then, in future, the cases for operation are properly selected, we may expect that the mortality will reach a reasonably low figure. I think we must agree with Fritsch in his statement that he has "no doubt that in the near future the mortality in general will come down to three or four per centum."

It certainly seems remarkable and far surpassing all expectations that, in less than ten years after the re-introduction of this operation in surgery, its mortality should have come down to almost the same point as that from laparotomy for ovarian tumors. Ovariotomy required a much longer series of years to attain the point of safety which it has now gained. Vaginal hysterectomy for carcinoma may now be said to be not more dangerous than the extirpation of a carcinomatous mamma, with removal of the axillary glands, for which operation Billroth in 1880 gave a mortality of ten per centum, a per centage still further reduced, as shown by Schmidt, from Küster's service in the Augusta Hospital in Berlin, to five and two-tenths per centum.

The partial operation of low or high vaginal amputation of the cervix has been urged as a substitute for vaginal hysterectomy in all cases of carcinoma of the vaginal portion, and the more limited cases of carcinoma of the cervix. The former operation is said to be much less dangerous than the latter. We must then ask the question: Are partial operations on the carcinomatous uterus, provided they permit of the effective removal of the tumor, preferable, as being much less dangerous than the operation of total extirpation?

Pawlick reports a large series of galvano-caustic operations from Braun's clinic in Vienna; one hundred and thirty-seven cases, with ten deaths—that is, seven and one-third per centum. In twelve of the cases of recovery late hæmorrhages of a severe character were observed. Schröder reports one hundred and thirty-six amputations of the vaginal portion and cervix, with ten deaths from sepsis—that is, seven and two-fifths per centum. Wallace reports ten cases with two deaths, or twenty per centum. Gusserow has had thirty-three cases, with three deaths, or nine per centum.

We may thus conclude that if the mortality of total hysterectomy is in the neighborhood of ten per centum the operation is not much more dangerous than the partial vaginal operation, and agree with Schatz, who wrote in 1883: "The danger of the high vaginal amputation does not seem to be much smaller than that of the total extirpation."

A much larger field than hitherto will undoubtedly be accorded in future to vaginal hysterectomy. It is in many cases almost impossible to determine, during the operation, how far up into the wall of the uterus the carcinomatous tissue extends. Even with a mortality of twenty-five per centum from total extirpation, Gusserow, in doubtful cases, prefers this to the partial operations. In this connection he states: "The safer the total extirpation becomes, the more will it take the place of amputation of the cervix."

The experience in a very complicated case of Binswanger is of importance in our choice of operation. He found a perfectly isolated portio-carcinoma, accompanied by an also isolated carcinomatous degeneration of the mucous membrane of the fundus. It is generally accepted as a law in surgery of the mammary gland that, however localized, a small carcinomatous nodule may be, nothing less than the removal of the entire gland,—and I think it safe to add, the lymphatics of the axilla,—would be the safe operative procedure to adopt. It can thus be understood that authors who believe in a low mortality for vaginal hysterectomy, as Sänger, Leopold, Fritsch, require this operation to be done in all cases of limited carcinomas, even small carcinomas of the vaginal portion, to the exclusion of any of the partial operations. Fritsch calls attention to the fact that a strict line of demarkation, between carcinoma of the cervix and of the vaginal portion, as described by Ruge and Veit in their classical article on "Uterine Carcinomas," does not exist in all cases. Some apparent portio-carcinomas extend deeply up into the cervix. It is often impossible, during a partial operation, to determine whether we operate in healthy tissue or not; consequently, in the majority of such cases, total extirpation is safer as a radical cure of the carcinoma than partial operation.

*Indications.*—It is important to determine in a given case whether a carcinoma can be operated upon with success, or if it should be let alone. Cancerous invasion of any of the tissues surrounding the uterus is a contra-indication for an operation. Especially important is the mobility of the organ. Impaired mobility usually means invasion of the broad ligaments, and to this class belong most of the cases in which the operation has had either to be abandoned or has been exceedingly difficult and dangerous, and been followed by speedy relapse in loco. If the uterus is not fairly movable, the technical difficulties of the extirpation are well-nigh insurmountable. At the same time we must be aware of the possibility of meeting with commencing carcinoma in a uterus immobilized by independent parametritic exudates. If in such a case we are able to make a diagnosis, there is the possibility of enucleating the uterus from the connective tissue masses in which it is imbedded, without opening the peritoneal cavity,—the so-called extra peritoneal vaginal hysterectomy. This operation was performed years ago by Czerny, and it has been lately recommended by Frank. As the cases up to this time are so few, and the descriptions of them so incomplete, further experience is needed.

Recently, vaginal hysterectomy has been resorted to in other diseases of the uterus than malignant tumors. Besides a not inconsiderable number of operations for prolapse of the uterus, Leopold has made the extirpation for severe neurosis; Bernays, Frank and others for retroflexion, and Frank in several cases of chronic granulating adenomatous endometritis, when complicated by hæmorrhage or parametritis. As yet the last named as indications for the operation do not seem to be generally accepted.

The *operation* itself, as far as the details of the procedure are concerned, is remakably unsettled, and it is difficult to find two surgeons who operate exactly in the same manner. The principal object to be attained in the choice of operation is security against hæmorrhage and sepsis of the abdominal cavity as well as of the vaginal wound. The more recent changes in the operation in this direction have undoubtedly contributed to lessen its immediate mortality.

The same preparatory measures are generally adopted as for laparotomy, with a view of having the alimentary canal empty. Thorough disinfection of the vagnia is, in the case of an ulcerating carcinoma, rather difficult, and opinions are divided whether the removal of the fetid carcinoma-tissue, with the sharp spoon, should take place a day or two before the operation (Hegar), or whether it can be postponed so as to be the first step in the operation. (Leopold & Fritsch.) It seems most convenient to do all the operating at once, when, as has been demonstrated by the two last named operators, sufficient immediate disinfection can be had during the operation.

The lithotomy position is the one almost exclusively chosen as the most convenient. Only a few, such as A. P. Dudley, of New York, prefer to operate in Sims' position. Mundé tried this position, but gave it up as inconvenient. It would seem that blood and irrigation-fluid would be more likely to enter the abdominal cavity, in Sims' position, on account of the difference in pressure in the vagina and abdominal cavity, characteristic of the latter position.

It will be necessary to divide a narrow vagina posteriorly, to make room for the operation. Simon's specula, or retractors of different sizes, or Fritsch's speculum with an apparatus for constant irrigation may be used.

It does not matter whether the uterus is drawn down into the vulva with a strong loop of silk or the forceps, provided the cervical tissue has consistency enough to give a good hold. Mikulicz's loop of silk through the lower part of the parametria does not help materially in bringing the uterus down, although it may assist in making the division of the lateral attachments bloodless. Fritsch lays great stress on commencing the operation by detaching the parametria from the sides of the cervix, which he accomplishes in the following manner: He divides the parametria after successive ligatures *en masse* of portions of the tissue, each one deeper than the preceding one, until after three to five such ligatures the lower border of the lateral ligament is reached. Hæmorrhage from the uterine artery or its branches is thus prevented almost entirely, and the uterus becomes loose, and can very easily be brought down into the vulva so as to give greater facility to the succeeding steps in the operation.

In the detachment of the bladder and rectum from the cervix, Martin and Hegar try to avoid hæmorrhage from the vagino-peritoneal wound surface by a row of sutures, including successively one portion after another of the tissues to be divided. This procedure takes a good deal of time, some of which may, however, be saved by not having to ligate bleeding points after the division, hæmorrhage from which, although not copious, may sometimes prove very troublesome.

The opening of the peritoneal cavity, before and behind the uterus, should always be preceded by a renewed, thorough disinfection of the entire field of operation. Right here arises the question of keeping septic material out of the peritoneal cavity. If all hæmorrhage is stopped by the above mentioned precautions, sepsis could only arise from contact between the peritoneum and the carcinomatous cervix and uterine canal. Fritsch recommends that the posterior fornix be left in connection with the uterus until the very last—that is, until after the detachment of the bladder and the ligature of the broad ligaments. A disinfected sponge is passed up between the uterus and the bladder and placed above and behind the fundus. To the sponge should be attached a loop of silk or silver wire (Leopold), as difficulty has sometimes been experienced in finding and removing a loose sponge from the pelvis. Fritsch draws down the fundus of the uterus through the anterior cul-de-sac while he ligates the broad ligaments, thus leaving the carcinomatous cervix in the posterior fornix, which has not yet been opened, and avoids contact with the peritoneum by making the division of the posterior fornix the last step in the detachment of the uterus.

Leopold opens into Douglas' fossa before ligating the broad ligament, but he never turns—that is, ante- or retrovertes the uterus. During the ligation of the ligaments the uterus is left in place, so that the carcinomatous cervix remains down in the vagina during the entire operation.

I have some doubt whether it is convenient or even possible in all cases to keep the uterus in situ during the ligature of the broad ligament. In my first case, in which the fundus was small, it was natural and easy. In the second case, in which the uterus was larger, I found it more easy to antevert. Thus Fritsch's advice in this direction appears more applicable to cases in general.

*Ligature of the broad ligaments* is one of the most important steps in hysterectomy, inasmuch as the large vessels, uterine and spermatic, must be ligated with entire safety so as to avoid fatal hæmorrhage. Fear of not thus securing the vessels from bleeding has brought forth a great variety of methods to attain this end.

The use of the galvano and thermocautery, to insure security against hæmorrhage from the broad ligaments, has been occasionally tried by Anderson and Simpson, and speedily abandoned as unsafe; Simpson's patient died from secondary hæmorrhage.

*Ligature* EN MASSE has been used by Billroth, Wölfler, Mikulicz, Schröder and Thiersch. The dangers of the ligature *en masse*, from its liability to slip, compelled Billroth to compress the ligament with strong forceps before applying the ligature. Schrödei added several separate ligatures. Ohlshausen used a Cintrat's wire constrictor, and, later, elastic ligature.

*Ligature in portions.*—Czerny had already ligatured in three portions, and many operators followed this method.

*Homostatic forceps* left in place for 24-36 hours. Pean.

Partial ligature, step by step, is probably the safest, and now the most commonly adopted method. It is hardly necessary, as Hofmeyer proposed, to divide the ligament between successive double ligatures, as there is very little hæmorrhage from the uterine ends of the vessels. Thus the single step ligatures, as proposed by Sänger, and now used by Martin, Von Teuffel, Fritsch, Leopold, and many others, is at present regarded as the safest method of procedure.

*The treatment of the peritoneal and vaginal wound* is of paramount importance, inasmuch as thereupon depends the prevention of septic inflammation in the peritoneal cavity, as well as in the tissues within the pelvis. Great diversity of opinion still prevails as to the best method to be adopted.

Schatz procures drainage by keeping the patient in a sitting position during the after-treatment—a method altogether inconvenient for the patient.

Schröder has left both wounds open; united the broad ligaments with the lateral corner of the vaginal wound, and placed a T shaped drainage-tube in Douglas' fossa.

Ohlshausen uses a drain in Douglas' fossa and iodoform-gauze in the vagina.

Martin uses a T shaped drain in

Douglas' fossa. Bottini, Simpson, Thiersch, Leopold, and Fritsch use iodoform-gauze drain from Douglas' fossa down into the vagina.

The peritoneal wound has been partially united and an opening into the peritoneal cavity left for drainage, either in the center or at each corner of the wound (Czerny). The peritoneal wound has been left open and the vaginal wound partly united (Sänger); iodoform, gauze in the vagina, the peritoneal woun d united and the vaginal wound left open (Billroth, Wolfler, Mikulicz, Kaltenbach, Von Teuffel.

The peritoneal wound has been united and the vaginal wound partly closed (Tauffer). Regarding this, Tauffer makes the following remarks: "I am convinced the total or partial leaving open of the vagino-peritoneal wound represents a stage in the development of vaginal hysterectomy which will soon be passed by, and that the total closure of the wound by suture will be generally adopted."

In the most cases of vaginal hysterectomy no wound-surfaces are left in the peritoneal cavity, and consequently there seems to be nothing to drain. When the ligatures in the broad ligaments are brought out into the vagina and kept there by sutures, there seems to be less reason to drain after vaginal hysterectomy than after uncomplicated cases of ovariotomy or oöphorectomy. In one of my cases (Case I.) total closure of the wound was followed by a perfectly aseptic course of after-treatment.

*Shall the ovaries and tubes be removed, or can they be left?*—In the majority of vaginal hysterectomies, as yet on record, the ovaries and tubes have been left, and yet complaints of sufferings from periodical disturbances in these organs are of very rare occurrence. Schröder and Tauffer report one case each, in which pain afterward might be referred to the ovary. Schröder's patient had pain at the time of her menstrual period, but there was never any hæmorrhage. Tauffer's patient had pain in an ovary, supposed to be included in the cicatrix.

Fritsch advises that the ovaries and the tubes be removed when the patient is young, and the organs can be brought down in the wound with reasonable ease. Leopold gives similar advice. Brennecke is of the opinion that the ovaries and tubes can always be left without any disadvantage to the patient. The ovariës, left in, seem in a short time to become atrophic and cease to have any physiological activity. He saw, quite commonly, especially in younger patients, more or less vague menstrual molimina in the first two to six months after the operation, but after that time they disappeared entirely. There would thus seem to be no reason to complicate hysterectomy, which in itself is a sufficiently long operation, by removal of the uterine appendages, except in cases where these are found to be diseased.

The *mishaps* of the operation seem to become more and more rare, because the advanced cases are not operated upon, and because the requirement that the uterus shall be freely movable is more strictly adhered to. Thus the difficulties in separating the uterus from its surroundings are the more easily overcome.

*Opening into the bladder* still not unfrequently occurs, even in the hands of the most skillful operators (Czerny, Ohlshausen, Fritsch, Martin, Hofmeyr and others). The opening, however, has usually no more serious consequences than the existence of a temporary vesico-vaginal fistula, which generally closes spontaneously, in a few weeks.

*Injury to the ureter.*—It was a common occurrence in the abdominal hysterectomy of Freund that one or both ureters were either divided or ligated. In the vaginal hysterectomy, injury to the uretur is just as rare as injury to the bladder is common. Starck, in 1882, had to remove a piece of the ureter, because it was involved in carcinomatous tissue. Bœckel, in 1884, had an injury to the ureter, probably from its being included in a hæmostatic forceps, which, according to Pean's method of operating, was left in permanently. In both cases subsequent nephrectomy was resorted to successfully. In early operations with a movable uterus, there is in the modern method of operating little or no danger of injuring the ureter during vaginal hysterectomy.

*Permanent recovery*, after vaginal hysterectomy for carcinoma, depends, as in all other removals of malignant tumors, partly on the anatomical characteristics of the latter, and partly upon early operation. The reports from the literature in this regard are imperfect, because the operation is new and the reports recent, and a number of patients have been lost sight of or their later condition has never been reported.

If we consider freedom from relapse, two years after the operation, as a permanent recovery,—a view that is generally adopted,—it is almost astonishing to see a percentage of permanent recovery amounting to 39.2 per centum, as published by Mundé in a select series of cases, which included only cases of early operation, eighty-two in all, with thirty-two permanent recoveries. Still more favorable are the reports published by Leopold, which give more than two-thirds, or sixty-nine per centum of radical cures.

We are certainly not accustomed, from other fields of surgery, to expect so beneficial results from operations for malignant tumors. Is this because carcinomas of the uterus, in most cases, belong to the benignant variety, or is it that the uterus, being a comparatively well isolated organ, gives favorable chances for operating in healthy tissue, by keeping the tumor localized for a certain length of time?

Should further reports coincide with the foregoing statistics, it seems evident that vaginal hysterectomy, for carcinoma, gives better chances for the patient than operations for carcinoma in any other part of the body.

Case I. Mrs. E. C. M., 28 years of age, married, residing in Chicago, came to my office on June 10, 1887. Her mother died of cancer of the stomach. The patient commenced to menstruate at the age of 15, and was always regular until the time of her marriage, in October, 1886. After that time the menses became somewhat irregular, and shortly afterward some leucorrhœa appeared. The discharge gradually increased, and, in the spring of 1887, became of a somewhat fetid odor, which caused her to apply for treatment. In January, 1887, the menstrual flow was excessive, and in February she was examined by Dr. S. D. Jacobson, of this city, who kindly informed me of the local condition of the patient. He found on the posterior side of the vaginal portion of the uterus a flat erosion, half an inch long and a quarter of an inch broad, surrounded by five or six small nodules looking like ovula Nabothi. In narcosis the erosion was scraped off with the sharp spoon, and the bleeding surface cauterized with a mixture of equal parts of chloride of zinc and alcohol. For two months it looked as if the loss of substance would heal up, but in May, a small, cauliflower-like excrescence appeared upon the surface.

On examination, I found the following condition: The patient is pale, but not emaciated. She seems to be in good health, except for the discharge from the vagina. Examination per vaginam shows the right half of the vaginal portion of the uterus to be the seat of a hard nodular mass. Extirpation of the carcinoma was agreed upon, the method of operating being left undecided. If vaginal amputation of the cervix proved to be insufficient for the removal of all the diseased tissue, then total extirpation of the uterus would have to be resorted to.

On June 14, the patient entered Emergency Hospital. She was prepared for the operation in the usual way, by being kept on liquid diet for several days, by thorough disinfection of the vagina by antiseptic injections, and by shaving of the external genitals.

On June 17, I operated, in the presence of Drs. Jacobson, Hall, Senior and Junior, Engert, Otto and Holmboe, in the following manner: The patient was placed in lithotomy position, and the vagina held open by Simon's specula. The narrowness of the vagina necessitated bilateral incisions in its posterior walls. The healthy part of the vaginal portion was seized with heavy vulsellum forceps, and the uterus drawn down toward the vulva. A loop of heavy silk was passed through a fold of the lateral fornix, through the lower part of the broad ligament, an inch outside of and above the vaginal portion, and knotted, with a view of securing the uterine vessels, and as a help to the further drawing down of the uterus. A circular incision was now made half an inch outside of the carcinoma and the vaginal portion. The parametrium or lower part of the broad ligament was divided without much hæmorrhage, each bleeding vessel was secured by forceps, and ligated with catgut. The uterus could now be brought down into the introitus. The carcinomatous tissue was scraped off from the ulcerated surface with a sharp spoon, leaving a cavity in the right posterior half of the cervix extending upward close to the internal os.

It was therefore thought advisable to remove the uterus in toto. Thorough disinfection of the cancerous cavity and the vagina was now made with 1:1000 corrosive sublimate solution. The dissection between the uterus and bladder was very easily made with blunt instruments, and finally the peritoneum was divided in the vesico-uterine fossa. From this opening, a flat aseptic sponge was passed up in the small pelvis, and a loop of heavy silk was passed through the lower part of the body of the uterus, to furnish a better hold on the latter than the vulsellum forceps could give. Dissection between the cervix and rectum was next made with blunt instruments, and the peritoneum divided. The remainder of the left broad ligament was secured by hooking the left index finger around it, and transfixed by a double silk ligature. It was then divided between this and the uterus without turning the body of the latter down into the vagina.

All visible divided vessels were now ligated with catgut, the right broad ligament was treated as the left had been, without difficulty, and the uterus was removed. The aseptic sponge originally inserted was now replaced by a larger one, which kept the omentum and small intestines out of sight. The cut ends of the broad ligament were drawn down into the vagina, and united by sutures to the lateral wall of the cervix. As the ovaries and tubes did not come down easily into the vaginal wound, it was decided to leave them in. Sutures were now passed antero-posteriorly through the vaginal wall of the anterior cervix, the posterior flaps of the peritoneum, and finally out through the posterior vaginal wall into the vagina. Six such sutures were, for the time being, left long, and secured by hæmostatic forceps. Before knotting these sutures, the flat sponge was removed from the pelvis, and the toilet of the pelvic abdominal cavity made by smaller sponges held by long artery forceps, until they came out dry and bloodless. Finally, the pelvis was iodoformized by means of a sponge dusted over with iodoform. The wound-surfaces between the vagina and peritoneum were then thoroughly cleansed with 1:4000 corrosive sublimate solution, and iodoformized. The vaginal sutures were now knotted, thus closing hermetically at the same time the peritoneal cavity and the vaginal wound. The ends of all sutures and ligatures were cut off an inch and a half from the knot. The vagina was irrigated with 1:1000 corrosive sublimate solution; dusted over with iodoform; and a light pad of iodoform-gauze was left in the vagina. The posterior incisions in the introitus of the vagina were united with sutures and covered with iodoform-gauze dressing.

The operation lasted two hours, at the end of which time the pulse was 90, and strong. There were no signs of collapse whatever. The course of the after-treatment was perfectly aseptic. There was no rise in temperature nor pulse, and no pain. For the first few days there was a little soreness in the region of the wound. After slight vomiting from the ether, the patient's appetite improved rapidly, and the first passage from the bowels, on the sixth day, was entirely painless. She sat up at the beginning of the third week, and the ligatures of the left broad ligament were loosened, and removed on the twentieth day. On the right broad ligament they still remained attached to the mortified peripheral end of the ligament, which was not detached until a week later. On the patient's discharge from the hospital, July 13, the transverse linear wound was completely closed.

Case II. Mrs. M. McE., aged thirty, was admitted to Emergency Hospital, June 14, 1887, and came under my care. She gives the following history: Parents living, no hereditary disease in the family. She was married in 1878, and has five children. Menstruation commenced at the age of fourteen, and was at times irregular until she reached the age of twenty, when it became normal. In the spring of 1883 she had an attack of pneumonia, and was then told by her attending physician that her uterus was out of place. For this she has at times been treated, ever since.

Early in 1886 she was informed by her physician that there was an ulceration at the neck of the uterus. A bad-smelling discharge commenced at about the same time. Until the beginning of 1887 she had no pain. In the summer of 1886 she became pregnant, and was delivered of a female child March 23, 1887.

She has had a cystic goitre the size of a fist for many years, which has never caused her any serious difficulty. For the last four years she has been in poor health, suffering from repeated attacks of bronchitis, and has gradually become emaciated.

*Present Condition.*—The patient is pale, considerably emaciated. A fluctuating tumor the size of a child's head takes up the anterior aspect of the neck from the hyoid bone to the sternum. It is tense and somewhat tender, as a result of puncture and aspiration undertaken in a hospital in the city, about a week before. The tumor is apparently superficially fluctuating. The tenderness is most pronounced around the seat of the puncture. Examination of the thoracic organs shows nothing abnormal. She has some cough, especially on rising, and raises a moderate amount of muco-purulent matter.

There is a discharge from the vagina of considerable quantity and fetid odor. Digital examination shows that the vaginal portion is transformed into an irregular, hard, nodulated mass, in the center of which the finger passes into a large irregular cavity, extending through the entire neck. The uterus is movable; the rectum and bladder do not appear to be infiltrated. There is no appreciable thickening in the broad ligaments. Pulse 110, and rather weak; temperature is normal; there is shortness of breath after exercise.

After the usual antiseptic preparations, I operated on June 30, 1887, in the presence of Drs. Jacobson, Rosa Engert, Otto, Hall, Sr., and Jr., Guerin Holmboe, and Simons. The vagina haa to be enlarged posteriorly; the uterus was drawn down with vulsellum forceps, and the same method of procedure pursued as in the previous case. The separation of the neck of the bladder from the uterus was difficult, because the carcinoma had extended into close proximity to the wall of the bladder, some of the muscular tissue of which was removed—so much so, in fact, that a sound in the bladder was covered by mucous membrane only. The neck was amputated at the internal os, because in this instance it proved more convenient to evert the body of the uterus through the opening in Douglas' fossa. The peritoneal wound was closed by a row of sutures, but the vaginal wounds in the lacunæ were left open in the middle for iodoform-gauze drainage. There was only slight hæmorrhage during the operation, which lasted more than two hours. Toward the end of the operation the patient was very much collapsed, cold and almost pulseless. In spite of the free use of hypodermic injections of brandy from the end of the operation at 2 P. M. until 8 P. M., no reaction had taken place; the pulse of the wrist was 160 and hardly perceptible, and the patient was a little delirious; temperature 96° F. I then made a transfusion in the median vein of the arm of twelve ounces of a $\frac{6}{1000}$ solution of salt, in sterilized water. The pulse improved somewhat after the transfusion.

June 21, 8 A. M. The patient was uneasy all night. She slept very little if any; pulse very feeble and can hardly be counted; complains of extreme thirst, has not vomited. 8 P. M. Condition unchanged. June 26. The patient's condition has gradually improved a little. She coughs considerably, and expectorates muco-purulent matter. Complains of frequent and painful micturition. Examination of the urine reveals cystitis, probably caused by a non-disinfected catheter used by one of the nurses. The bladder was washed out twice a day with a saturated solution of boracic acid. June 30. There is some bloody discharge from the vagina. July 15. Patient has been steadily improving; cystitis and cough less; she sits up, is out of bed, and eats and sleeps well.

July 20. Patient complains of considerable pain in the goitre, with some difficulty in breathing—so much so that aspiration was deemed necessary and a pint of greenish fluid evacuated, containing numerous, cholesterine crystals, pus-cells and blood-corpuscles. August 1. Sutures and ligatures were removed from the vagina, and the fornix presents a healthy granulating surface, an inch and a half broad and three quarters of an inch in antero-posterior diameter. The patient left for her home.

Case III. Mrs. C.—— H——, of Chicago, 32 years of age, married, was admitted to Emergency Hospital on August 21, 1887, and came under my care. She gives the following history: She did not commence to menstruate until the age of 19; menstruation was always scanty, but not painful; she was married at the age of 21, and has one child, 11 years of age. Three years ago her husband deserted her. Nine months ago she went under a "specialist's" treatment for "ulceration of the womb." She did not at this time, however, suffer from any subjective symptoms referable to the genital organs. She has never had pain (slight occasional abdominal pains excepted) or hæmorrhage until shortly before her admission to the hospital. She noticed that, from the middle of last winter to this time, she has gradually lost in weight. She does not appear cachectic.

*Present Condition.*—Patient is somewhat pale, but reasonably well nourished. Heart and lungs are normal. Urine in quantity, and, on chemical and microscopic examination, normal. There is a bloody, serous fetid discharge from the vagina. Vaginal examination reveals an irregular, hard, nodulated mass, occupying the right half of the vaginal portion of the uterus. The granulated surface is about an inch in diameter, and shows a peripheral area of grayish-red, rough, granulated appearance, surrounding a central area of a light, yellowish-gray color. Above the tumor the neck feels normal, except posteriorly and to the right, where it is harder than usual. The uterus is movable; no thickening or hardening of the parametria. The ovaries are of normal size; the body of the uterus somewhat enlarged; the cavity three inches deep.

*Diagnosis.*—Carcinoma of the vaginal portion, with probable slight extension up into the neck. It was decided to perform total extirpation according to the method of Fritsch and Leopold.

On August 25, in the presence of Drs. Picard, Bernauer, Engert, Hall, Otto and Holmboe, I operated according to the method of Fritsch. The patient had been prepared for the operation in the usual way. The vagina, being very narrow, had to be divided posteriorly. Even after this had been done, the space was so small as to make the operation very difficult. The granulating surface of the carcinoma was removed by a sharp spoon, and the surface then cauterized with the ten per centum chloride of zinc solution. The cervical canal, being now fully exposed, was, together with the uterine canal, disinfected by means of cotton dipped in corrosive sublimate solution and iodoform. A ligature was passed through the parametria, the uterus drawn down with vulsellum forceps; the left parametrium incised to the extent of an inch, half an inch outside of the vaginal portion. The tissue of the left parametrium was now ligated in portions, and then divided between the ligature and the uterus. Four or five such ligatures were applied before the movable portion of the broad ligament could be reached by the index finger. The ligatures were left long, and the bleeding points and visible vessels between the ligatures ligated separately. The right parametrium was now dealt with in exactly the same manner. This part of the operation was almost bloodless. These steps in the operation had the effect of making the uterus come down more than an inch, so as to permit the vaginal portion to be well outside of the vulva. Notwithstanding this, the detachment of the bladder from the uterus was exceedingly difficult. A curved steel sound in the bladder marked its outlines clearly, and the anterior fornix was incised. An attempt to separate the bladder from the uterus with the finger and dissecting forceps proved utterly futile. Every particle of tissue had to be divided with scissors. An attempt to further the separation by most careful use of closed dissecting forceps resulted in making an opening into the bladder a quarter of an inch wide. This opening was immediately united by a continuous catgut suture, taking in only the muscular coat, and finally knotted in such a way as to draw the opening together like a tobacco pouch.

Finally, after having been obliged to leave in situ a thin layer of uterine tissue on the wall of the bladder, the peritoneum was reached and the vesicouterine fossa opened transversely on each side out to the broad ligaments. A good-sized flat sponge was passed up over and behind the body of the uterus, with a silk loop attached to it. Several vessels and bleeding points had to be secured on the vesical side of the wound. The left index finger was passed up behind the left broad ligament, and the latter ligated in three portions, and cut off from the uterus. The right broad ligament having been treated in the same manner, the uterus was now attached, posteriorly, only to the tissues between the posterior fornix and Douglas' fossa. This bridge of tissue was divided by scissors and secured by a loop. In endeavoring to remove the uterus there was found a number of long, band-like adhesions between the posterior surface of the uterus and the peritoneum in Douglas' fossa. Separation of these adhesions was followed by very troublesome, although not copious, hæmorrhage, a number of bleeding points having to be secured and ligated separately. The ovaries and tubes were left, as they were almost immovably fixed to the sides of the pelvis, but were otherwise of normal appearance. On the removal of the sponge from the pelvis, a mass of omentum and a loop of the small intestine came down into the vagina, and were pushed back by another disinfected sponge. The ends of the broad ligaments were secured in the corners of the vaginal wound in the usual manner, and the peritoneal flaps united carefully after cleaning the pelvis with sponges dusted over with iodoform. The center of the vaginal wound was left open on account of the bladder having been penetrated. The vagina was thoroughly disinfected, and, together with the vaginal wound, packed loosely with iodoform-gauze.

The after-treatment was entirely aseptic, and the patient was discharged from the hospital.

Case IV. Mrs. J. M., age 39, married, was admitted to Emergency Hospital September 6, 1887. The patient resides in Wisconsin. Her family history is good. With the exception of two attacks of typhoid fever in childhood, she has always had good health. Menstruation commenced at the age of 13, and has always been regular, but it was always accompanied by severe molimma. She was married at the age of nineteen, and has had four children, born respectively one year, three years, six years, and nine years after marriage. The last pregnancy terminated in a premature birth, the child living only a couple of hours.

The patient's health was always good until the time of her last pregnancy, eleven years ago. Since then she has had a white discharge from the vagina, and has been treated for "falling of the womb." She has never had pain. The first symptom to attract attention was the fact that the discharges became offensive, and of a darker color. This was first noticed in the spring of 1887. In other respects all went on as usual until July, 1887, when the patient had a severe hæmorrhage after her regular period. The hæmorrhages became more and more frequent, occurring almost every day.

*Present condition.*—Patient is fat, but well proportioned, weighing about 180 pounds. She is pale, but not cachectic. Her general health good; in fact, she would not have sought medical advice had it not been for the hæmorrhages, which alarmed her.

Vaginal examination reveals a nodulated, hard, ulcerating mass occupying the right half of the cervix. The uterus is movable. Right broad ligament rather tense, but not infiltrated. Rectum, vagina and bladder free.

*Diagnosis.*—Carcinoma of the uterus. Treatment: Vaginal hysterectomy.

After the usual preparatory treatment the operation was performed, September 12th. The patient was anæsthetized with ether and placed in lithotomy position. The introitus of the vagina was so narrow, from overabundant adipose tissue, that it was necessary to make a perineal incision on both sides. The carcinomatous mass was now scraped out with a sharp spoon, and the vagina thoroughly disinfected. The uterus was seized with heavy vulsellum forceps, and drawn down as far as possible. The broad ligaments were so short and unyielding that it was impossible to draw the uterus as far down as in the previous cases, making the operation very difficult throughout.

Bilateral incisions were made; the parametria ligated in portions, as usual, and the uterus removed, which was found to be unusually large. On account of the narrowness of the vagina, and the unyielding condition of the parts, the usual method of suturing and closing the wound was abandoned.

This course was the more indicated, as during the operation the patient's pulse was very rapid, and toward the end she showed signs of collapse. A rubber drain, surrounded by iodoform-gauze, was introduced, and ordinary antiseptic dressing applied.

At the end of the operation the patient was very weak, radial pulse scarcely perceptible, necessitating the free use of hypodermic injections of brandy and ether.

The after-treatment consisted of daily irrigation with boracic acid, fresh packing of iodoform-gauze, and free use of stimulants.

Four days after the operation the rubber drain was removed; but it was necessary to introduce it again two days later, as the discharge became highly offensive. It was kept in for two days, after which iodoform-gauze only was used.

From the beginning of the third week after the operation, the temperature and pulse became normal, and remained so, excepting one afternoon (October 6th), when she was allowed to sit up half an hour, after which her temperature went up to 102° F. She was kept in bed for several days, not being allowed to sit up again until October 13th. Since that time her recovery has been uninterrupted. All ligatures were removed, and the wound entirely closed. Her appetite and general condition are good.

269 La Salle Avenue.

LITERATURE.

Transactions of the American Gynæcological Society, Vol. 9, 1884, p. 195.

Paul F. Mundé.—The Proper Limitation of the Operation of Complete Vaginal Hysterotomy for Cancer of the Uterus.

A. Martin.—Pathologie und Therapie der Frauenkrankheiten. Wien, 1887, p. 304.

Klotz.—Centralblatt für Gynæcologie, No. 2, 1886.

T. Gaillard Thomas.—Medical and Surgical Reporter, March 7, 1885.

Fritsch.—Archiv. für Gynæcologie, Bd. 29, p. 359, Sechssig Totalextirpationen des Carcinömatösen Uterus.

Leopold.—Archiv. für Gynæcologie, Bd. 30, p. 401. Achtundvierzig Totalextirpationen des Uterus wegen Carcinome Totalprolaps und schwere Neurosen.

Billroth. — Krankheiten der Brustdrüsen Deutsche Churgie by Billroth and Luecke. Lieferung 41, p. 155.

Schmid.—Zur Statistic der Mamma Carcinome und deren Heilung. Deutsche Zeitschrift für Chirurgie, Bd. 26, p. 139.

Pawlick.— Behandlung der Uterus Carcinome. Wien, 1882. Vide Gusserow, Die Neubildungen der Uterus. Stutgart, 1885.

Hofmeyer. — Zeitschrift für Geburtshülfe und Gynæcologie, Bd. 13, II. Ueber die entgültige Heilung des Carcinoma Cervicis Uteri durch die Operation.

Wallace.—British Medical Journal, September 15, 1883.

Gusserow.—Die Neubildungen des Uterus. Deutsche Churigie, by Billroth and Luecke. Leipzig 57, p. 233.

Schatz.—Archiv. für Gynæcologie, Bd. 31, 1887, p. 409. Klinische Beiträge zur Extirpation des ganzen Uterus durch die Vagina.

Binswanger.—Centralblatt für Gynæcologie, 1887, p. 1.

Ruge und Weit. — Zeitschrift für Geburtshülfe und Gynæcologie, Bd. 2, p. 415.

Sänger.—Archiv. für Gynæcologie, Bd. 21, p. 29, zur. vaginalen Totalextirpation, 19.

Frank.—Archiv. für Gynæcologie, Bd. 30, p. 1. Ueber extraperitoneale Uterusextirpation.

Hagar und Kaltenbach. — Die operative Gynæcologie. Stutgart, 1886.

Tauffer.—Zur Trage der Totalextirpation des carcinomatösen Uterus. Archiv. für Gynæcologie, Bd. 23, p. 367.

Brennecke.—Ueber die vaginale Totalextirpation des Uterus. Zeitschrift für Geburtshülfe und Gynæcologie, Bd. 12, p. 56.

Storck—Berlin Blinre Wochenskrifpt, 1882, p. 12.

Boeckel.—Bullet dé la Société dé Chirurgie. Juine Juilliet vide. Virrchow Jahressber für 1884. Bd. 2, 1884, p. 626.

